# Nonlinear Dynamics of Thick Hybrid Composite Laminates Subjected to Low-Velocity Impact and Various Preloading

**DOI:** 10.3390/ma18102331

**Published:** 2025-05-16

**Authors:** Aiqin Tian, Chong Li, Long Ma, Xiuhua Chen

**Affiliations:** 1 School of Aeronautics and Astronautics, Shanghai Jiao Tong University, Shanghai 200240, China; tianaiq@163.com (A.T.); chenxiuhua@sjtu.edu.cn (X.C.); 2 National Engineering Research Center for High-Speed EMU, CRRC Qingdao Sifang Co., Ltd., Qingdao 266111, China; malong@crrcsf.com

**Keywords:** low-velocity impact, preloading, hybrid composite laminate, thickness, progressive damage model

## Abstract

The composite primary structures of railway vehicles endure not only mechanical loads including tension, compression, bending, and torsion, but also external impacts, such as by the crushed stone in ballast. In the present study, the low-velocity impact response of preloaded hybrid composite laminates with different thicknesses is examined using a finite element method based on a progressive damage model. The hybrid plate consists of carbon fiber-reinforced unidirectional and woven prepregs. The progressive damage model, based on the 3D Hashin model, is validated by experiments on hybrid laminate, and further compared with the post-impact appearance obtained from CT scans. Preloading, considered to be tensile, compressive, or shear, corresponds to different positions in a bending beam with flanges and a web. Finally, the effects of impact energy, preloading, thickness, and impact angle on the dynamic response are analyzed, with an emphasis on new results and failure mechanism analysis comparing the influence of preloads under a given impact energy and different thicknesses.

## 1. Introduction

In the rapidly evolving landscape of transportation technology, the integration of advanced composite materials [1] into vehicle design has become essential for enhancing performance, efficiency, and sustainability. Carbon fiber-reinforced polymers (CFRPs) have emerged as a revolutionary choice due to their exceptional strength-to-weight ratio, high stiffness, and excellent fatigue resistance when compared to steel and glass fiber-reinforced plastic composites [2]. Their application in track vehicles, such as high-speed trains and urban transit systems, represents a significant advancement in addressing the growing demand for lighter, more efficient, and environmentally friendly transport solutions. A shining example is the Qingdao Metro Line 1 train, developed by CRRC Sifang, which features the “World’s First Carbon Fiber Metro Train” with the body and bogie entirely made of composite materials.

The primary motivation for using CFRPs in track vehicles is the reduction of overall weight, which directly impacts fuel consumption and operational costs. Traditional materials like steel and aluminum, while robust, are often heavy and can limit design flexibility. CFRPs offer a lightweight alternative [3] that enhances energy efficiency without compromising structural integrity. This is particularly important in the context of reducing greenhouse gas emissions and promoting sustainable transportation. Research indicates that reducing the weight of the bogie by 25–40% leads to a decrease in vehicle operating energy consumption by over 15%, a reduction in wheel-rail wear by more than 30%, a noise reduction of 2–3 dB, and a decrease in the total lifecycle cost by over 15%.

However, one critical challenge in the application of CFRPs in track vehicles is their susceptibility to foreign object damage (FOD) [4,5]. Track vehicles often operate in environments where they can be exposed to impacts from debris, such as crushed stone in ballast. These impacts can cause delamination and other forms of damage, potentially compromising the structural integrity of the vehicle. Therefore, the design and manufacturing of CFRP components must incorporate robust impact-resistant features and regular inspection protocols to ensure safety and reliability, which includes the adoption of novel metamaterials [6,7,8], nano-fillers (CNTs [8,9] or graphene platelets [10,11]), and hybrid configurations.

A hybrid composite structure [12], for example combining unidirectional tape at its core with a two-dimensional woven fabric surface layer, offers a smart solution for boosting mechanical performance, streamlining production, and cutting costs. The unidirectional tape delivers exceptional strength and stiffness where it is needed most, while the two-dimensional fabric adds to the material’s in-plane strength, resistance to shear between layers, and surface finish. This clever design not only ensures long-lasting durability and lighter weight but also enhances overall performance, making it a top pick for high-speed trains and other rail vehicles. Not merely the combination of glass fiber reinforced composites and carbon fiber reinforced composites, as seen in [13], Wang et al. [14] carried out a series of experiments to investigate the impact of varying mixing ratios and stacking orders on the ability of carbon fiber/glass fiber unidirectional and woven hybrid laminates to withstand impacts and maintain their performance after being compressed. In a recent study [15], scientists explored how T800-SC carbon/epoxy laminates, made with Automated Fiber Placement (AFP) technology, hold up under low-velocity impact (LVI) and compression after impact (CAI). They compared two types of hybrid laminates—one made with unidirectional layers and another with pseudo-woven mesostructured composite (MAC) layers—against the standard quasi-isotropic laminates. Moreover, using mold pressing technology, researchers [16] have developed a biomimetic hybrid laminate that blends the strengths of both unidirectional and woven fiber arrangements. They then thoroughly investigated how well this innovative material can withstand impacts and resist damage.

Moreover, impact simulation is a crucial tool for assessing how well composite materials can withstand impacts. When it comes to analyzing damage in composites, the Hashin criterion [17] is a go-to method. It is great at predicting how these materials will fail under various types of stress. The Hashin criterion works by identifying four distinct ways materials can break down. To simulate this, researchers often use the finite element method (FEM) paired with a progressive damage model. As the simulation progresses, the Hashin criterion continuously updates the material’s damage status until it finally fails. This approach not only gives a precise picture of how damage develops during an impact but also helps evaluate how factors like thickness and ply characteristics [18] affect the material’s performance. Liu et al. [18] demonstrated that their advanced finite element model (FEM), incorporating the VUMAT material model, effectively captures the step-by-step failure of carbon fiber-reinforced composite corrugated plates during quasi-static compression. Moreover, their findings highlight that structures with a [0/90]s layup angle excel in energy absorption, making them a top choice for such applications. When pitted against the built-in 2D model in ABAQUS 6.14 and later versions, the 3D VUMAT subroutine proposed by Zhang et al. [19] comes out on top in terms of precision and performance. This breakthrough highlights the subroutine’s standout capabilities in modeling the mechanical behavior and failure processes of fiber-reinforced polymer (FRP) materials. Wang et al. [20] investigated how T700 carbon fiber-reinforced BA9912 composite materials behave when subjected to low-speed impacts, focusing on their damage and failure mechanisms. Shi et al. [21,22] modelled damage evolution in composite laminates subjected to low-velocity impact, in which the nonlinear shear behavior of the composite was described by the Soutis shear stress–strain semi-empirical formula.

In rail vehicles, composite components like side beams experience mechanical forces while in use. Depending on where an impact occurs, these forces can act as pre-stresses. For example, in a rectangular beam that is bending, the top and bottom surfaces will either stretch or compress, while the middle section (the web) will experience shear forces. Understanding how these pre-stresses affect the beam’s response to impacts is crucial for real-world engineering applications. As has been pointed out by Ji et al. [23], when composite laminates are under compressive pre-stress, their ability to resist impact damage decreases, which can negatively affect their overall damage tolerance. This means that when testing structures designed to handle compressive loads, it is crucial to account for how this pre-stress might impact their resistance to damage from impacts. Lan et al. [24] used a high-speed gas gun to perform a series of ballistic experiments. Their goal was to understand how applying a biaxial in-plane tensile preload influences the delamination of carbon fiber-reinforced polymer (CFRP) laminates when subjected to high-speed impacts. Langella et al. [25] carried out a series of experiments to explore how tensile preload influences the behavior of thin woven composite laminates when they are subjected to impact. To achieve this, the team employed a custom-built apparatus that applied uniaxial force to the test samples. Low-velocity impact on preloaded and curved laminates was investigated by Panciroli et al. [26], who concluded that more attention is required in the design and maintenance of preloaded and curved laminated composite structures.

However, there are no specific studies in the existing literature that compare the differences between thick and thin (typically < 5 mm) plates. In the present study, the low-velocity impact response of preloaded hybrid composite laminates with different thicknesses (4 mm and 30 mm) is examined using finite element methods (FEM) based on a progressive damage model. The hybrid plate consists of carbon fiber-reinforced unidirectional and woven prepregs. The progressive damage model, based on the 3D Hashin model, has been validated by experimental tests on hybrid laminates. Preloading, considered to be tensile, compressive, or shear, corresponds to different positions in a bending beam with flanges and a web. The effects of impact energy, preloading, thickness, and impact angle on the dynamic response are analyzed, with an emphasis on comparing the influence of preloads under a given impact energy and different thicknesses.

## 2. Methods

**Figure 1:**
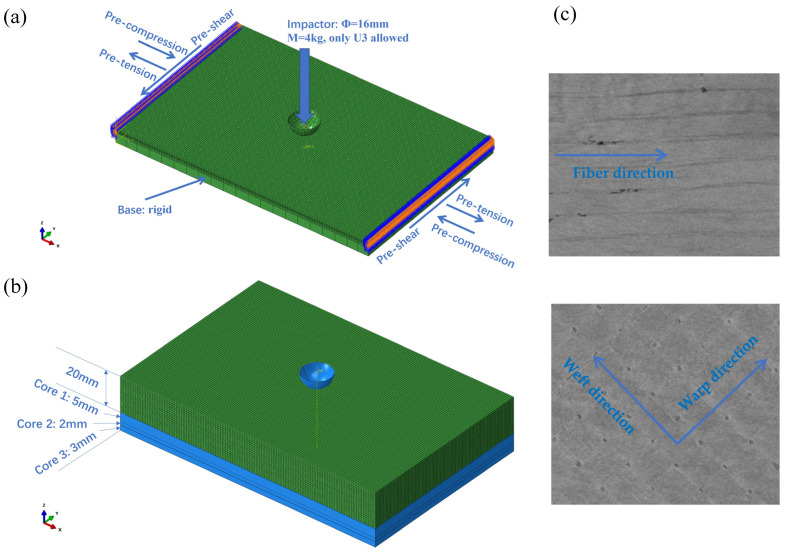
Laminated plates: (**a**) loadings with thin plate as example; (**b**) sublaminates of thick plate; (**c**) schematic of UDLM and WOVN layers from our CT scan images.

The laminates considered in this paper include unidirectional and woven layers, as shown in Figure 1, denoted by UDLM and WOVN in the modeling. Firstly, the constituent materials are named as those. In the user subroutine VUMAT, the relevant code is triggered when the material is labeled as either “UDLM” or “WOVN”. The failure criteria include [21,22]:

(1) $${\left(\frac{\sigma_{1}}{X_{t}}\right)}^{2}+{\left(\frac{\tau_{12}}{S}\right)}^{2}+{\left(\frac{\tau_{13}}{S}\right)}^{2}=1$$

in which *σ*_1_ represents the stress acting along the direction of the fibers, *X*_t_ denotes the tensile strength in the same fiber direction, *τ*_12_ and *τ*_13_ are the shear stresses, and *S* stands for the shear strength.

(2) $$(\frac{\sigma_{1}}{X_{c}})=1$$

where *σ*_1_ represents the stress acting along the direction of the fibers, while *X*_c_ denotes the material’s compressive strength in that same fiber direction.

(3) $${\left(\frac{\sigma_{2}+\sigma_{3}}{{2Y}_{t}}\right)}^{2}+{\left(\frac{\tau_{12}}{S}\right)}^{2}+{\left(\frac{\tau_{13}}{S}\right)}^{2}=1$$

in which *σ*_2_ and *σ*_3_ represent the stresses acting along the matrix direction, while *Y*_t_ denotes the matrix’s tensile strength.

(4) $${\left(\frac{\sigma_{2}+\sigma_{3}}{{2Y}_{c}}\right)}^{2}+{\left(\frac{\tau_{12}}{S}\right)}^{2}+{\left(\frac{\tau_{13}}{S}\right)}^{2}=1$$

where *Y_c_* denotes the matrix’s compressive strength.

The damaged stiffness matrix is taken as follows

(5) $$\left[\begin{array}{ccccccc} (1-d_{f})C_{11} & (1-d_{f})(1-d_{m})C_{12} & (1-d_{f})C_{13} & & & & \\ & (1-d_{m})C_{22} & (1-d_{m})C_{23} & & & & \\ & & C_{33} & & & & \\ & & & (1-d_{f})\left(1-d_{m}\right)G_{12} & & & \\ & & & & (1-d_{m})G_{23} & & \\ Sym. & & & & & (1-d_{f})G_{13} & \end{array}\right]$$

where *C_ij_* are the material coefficients or stiffness tensor, satisfies

(6) $$\sigma_{i}=C_{ij}\varepsilon_{j}+\sigma_{i}^{0}$$

in which $\sigma_{i}^{0}$ is the initial stress and will be zero when no preloading is applied.

Moreover, the *d_f_* and *d_m_* are the fiber and matrix damage, respectively, of which [27] the definitions are

(7a) $$\begin{array}{c} d_{f}=1-\left(1-d_{ft}\right)\left(1-d_{mt}\right) \end{array}$$

(7b) $$\begin{array}{c} d_{m}=1-\left(1-S_{mt}d_{mt}\right)\left(1-S_{mc}d_{mc}\right) \end{array}$$

where the *S_mt_* and *S_mc_* are coefficients to control the shear stiffness due to matrix damage and can be set as *S_mt_* = 0.9 and *S_mc_* = 0.9, as suggested by Zhou et al. [28].

In the initial stage of interlayer delamination, we employed cohesive elements (COH3D8) based on the quadratic stress criterion to simulate [29]. As for the propagation process of delamination, it was tracked using a mixed-mode propagation model [30,31].

The specimen is designed, according to ASTM D7136 [32], to have an in-plane dimension of 100 mm × 150 mm, while the thickness varies with the change in layups. For the thick plate, three layers of core material are positioned on the inner side of the main material with a thickness of 20 mm, as demonstrated in Figure 1b. The impacted plate is discretized using the 3D solid elements (C3D8R), of which the density is determined through a convergence analysis. The impact head features a hemispherical tip, with a total fixed weight of 4 kg, and the impact energy is controlled by adjusting its height. During the simulation, a hemispherical analytical rigid body was used to represent the punch, which was assigned a specific mass and initial velocity. To simulate the interaction between the punch and the impacted plate, general contact is defined with a friction coefficient of 0.3 [21,22]. This contact includes the outer surface of the punch and all surfaces of the impacted plate within the vicinity of the impact zone. This approach ensures a more accurate representation of the contact between the exposed surfaces and the punch following the impact failure.

**Figure 2:**
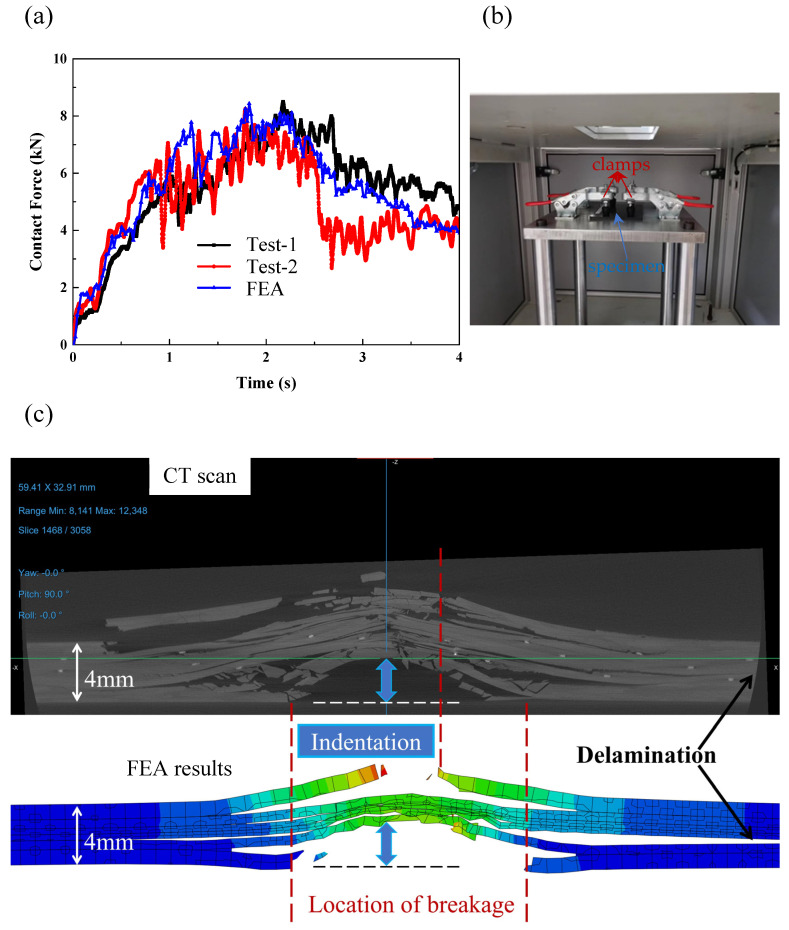
Comparison of simulation and test of the LVI of hybrid plate (Type-A): (**a**) the contact load–time curves; (**b**) the installation of specimen; (**c**) CT (upper) and FEA (lower) results of the mid-span section after impact.

We conducted the simulation analysis in two steps. In the first step, a preload was applied to the test specimen, for which an amplitude of smooth step is defined, establishing rigid contact with the rigid support base at the bottom. In the second step, the impact load begins to take effect while the preload was maintained, and fixed boundary conditions (defined by U1 = U2 = U3 = 0 for the 3D solid elements used) were defined in the four clamping regions to simulate the securing effect of the four clamps during the test, as shown in Figure 2b.

In this study, a Diondo X-ray microscope (Type d5) was employed to conduct X-ray CT scans. The scanning resolution was set at 20 μm. To reduce its size and increase the efficiency, the specimen was initially cut, remaining only on the area surrounding the impact site. During the analysis, X-rays were emitted from the source and penetrated the reduced sample. Subsequently, a series of two-dimensional X-ray images were reconstructed (by using Dragonfly) into a three-dimensional model. Since different materials exhibit distinct X-ray mass attenuation coefficients, this property was utilized to differentiate and visualize damage within the surrounding materials. Finally, we utilized the Dragonfly software (version 2022.2.0.1367) for model reconstruction and visualization, as illustrated in Figure 2c.

## 3. Results and Discussion

Two typical thicknesses are considered and compared, for which the layups are presented in Table 1. As indicated before, both consist of two kinds of polymer composites with matrixes of epoxy and reinforced by T700 carbon fibers (Zhongfu Shenying Carbon Fiber Co., Ltd., Jiangsu, China).

For the composite materials of UDLM and WOVN, denoting unidirectional tapes and two-dimensional fabrics, the material properties possessed are shown in Table 2, in which the 1 and 2 refer to directions along and perpendicular to the fiber in UDLM and warp and weft direction (refer to Figure 1), respectively. In addition, core-1, core-2, and core-3 are three groups of laminates considered to provide stiffness, for which the elastic properties are listed in Table 3. During the manufacturing process, these three core layers were prepared prior to the initiation of the 20 mm structural layup of the main body. In addition, the fracture energies of woven fabrics are assumed to be G1t = G1c = G2t = G2c = 11 N/m [33], while for the cohesive elements representing the interface between the lamina, the power exponent corresponding to the B-K criterion is 1.45. The cohesive strength is taken as 75 MPa for mode I and 45 MPa for mode II and mode III. Moreover, the value of toughness for mode I, II, and mode III are 0.7, 1.4, and 1.4 N/mm, respectively.

### 3.1. Comparison of Simulation and Test

Consider a scenario involving an impact with an energy of 50 joules. In this case, the velocity of the impact would be 5 m/s. The impacted plate has a nominal thickness of 4 mm and is constructed using a Type-A layup. As illustrated in Figure 2a, our finite element simulation results align well with the two experimental outcomes, demonstrating that our modeling approach is both effective and accurate. As illustrated in Figure 2c, the simulation results, including features such as fracture, delamination near the impact point, and the depth of the dent, show a high degree of consistency with the post-impact morphology obtained from CT scans.

### 3.2. Parameter Analysis

**Figure 3:**
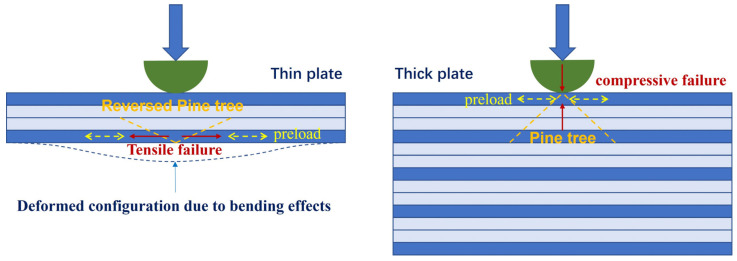
Comparison between LVI responses of preloaded thin and thick laminated plate.

In this section, we first compare the differences between the LVI response of thin and thick composite plates under preloading. Generally, the two have different ways in which they fail. As shown in Figure 3, when thicker composite laminates are hit, the intense pressure at the point of impact can cause tiny cracks to form on the surface. These cracks then grow downward, creating a pattern that looks like the branches of a pine tree. But in thinner laminates, things play out differently. Here, the bending stress causes cracks to start at the bottom layer first. From there, cracks within the layers and separations between layers spread upward, eventually creating a pine branch-like pattern on the surface, but in reverse [34].

#### 3.2.1. Effects of Preloading on Thin Plate (26.8 J)

This energy is determined according to ASTM 7136, which suggested to be 6.67 J/mm.

**Figure 4:**
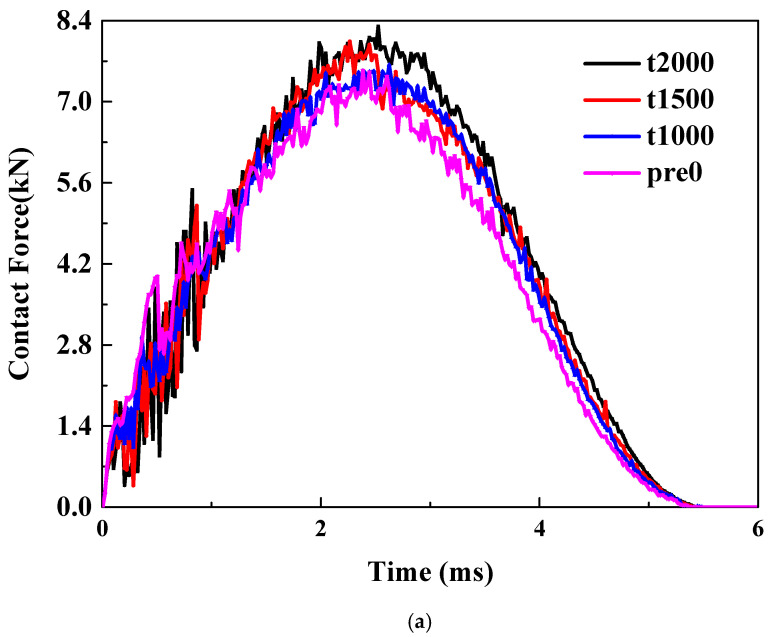

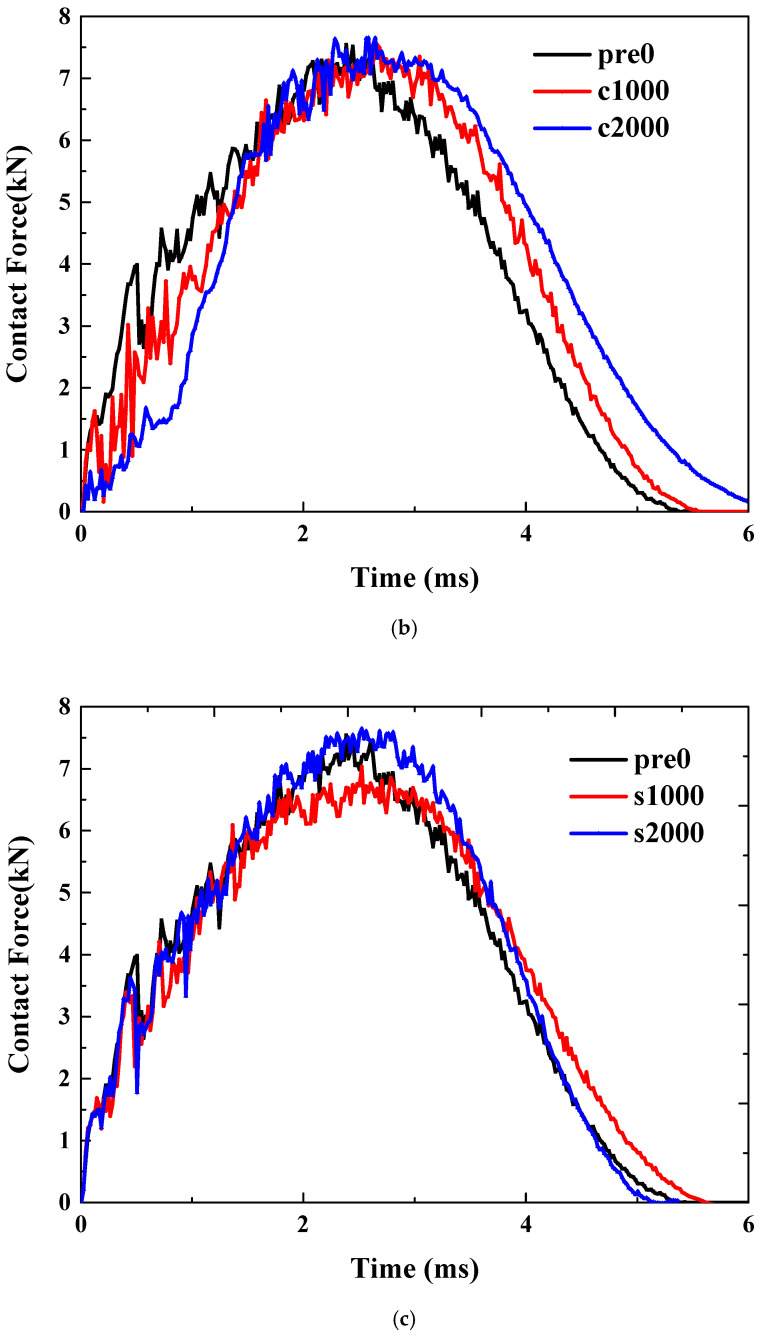
LVI response of thin plate under: (**a**) pretension; (**b**) precompression; (**c**) pre-shear.

Pretension. As illustrated in Figure 4a, the trend of the curve remains consistent as the tensile pre-strain increases from 0 to 2000 με. However, clear but not significant differences between the peak loads can be observed, indicating that the initial tensile load can enhance the impact force. The primary reason is that the tensile preload significantly boosts the laminate’s ability to resist bending.

Precompression. Unlike tensile preloads, when a compressive preload is applied, the curve exhibits a noticeable offset, as illustrated in Figure 4b. Compared to the scenario without any preload, the application of a compressive preload delays the occurrence of the peak force. Moreover, both the ascending and descending segments of the curve are offset backward. This is mainly because the compressive preload will intensify the bending degree of the material during the impact process.

Pre-shear. The application of shear preload introduces a more complex scenario. Compared to the case without pre-strain, applying 1000 micro-strains of shear reduces the peak impact force. However, as shown in Figure 4c, when a larger pre-strain of 2000 micro-strains is applied, the peak impact force increases. This indicates that the effect of shear preload is not monotonic; instead, it initially decreases and then increases the impact load. A specific amount of shear load can enhance the bending stiffness, resulting in a higher impact force. But when the shear load is limited, it causes more intense delamination, which caps any further rise in the impact contact force.

#### 3.2.2. Effects of Preloading on Thick Plate (100 J)

**Figure 5:**
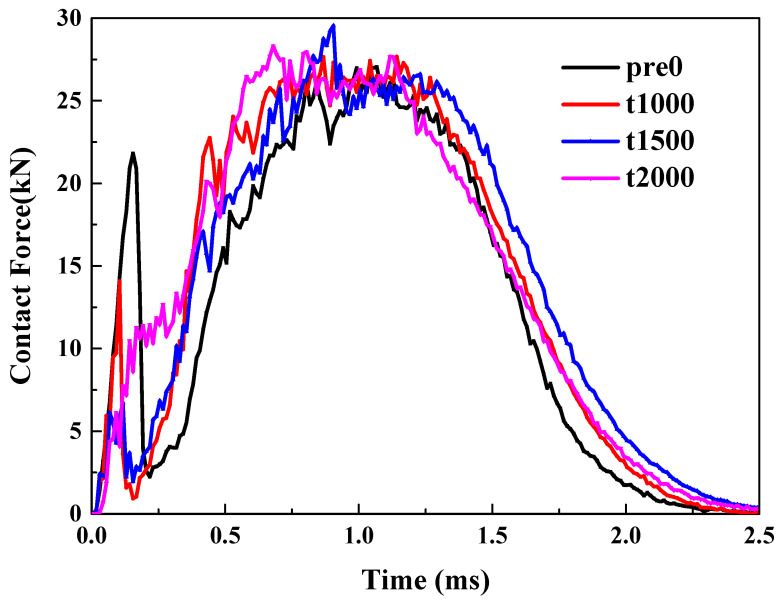

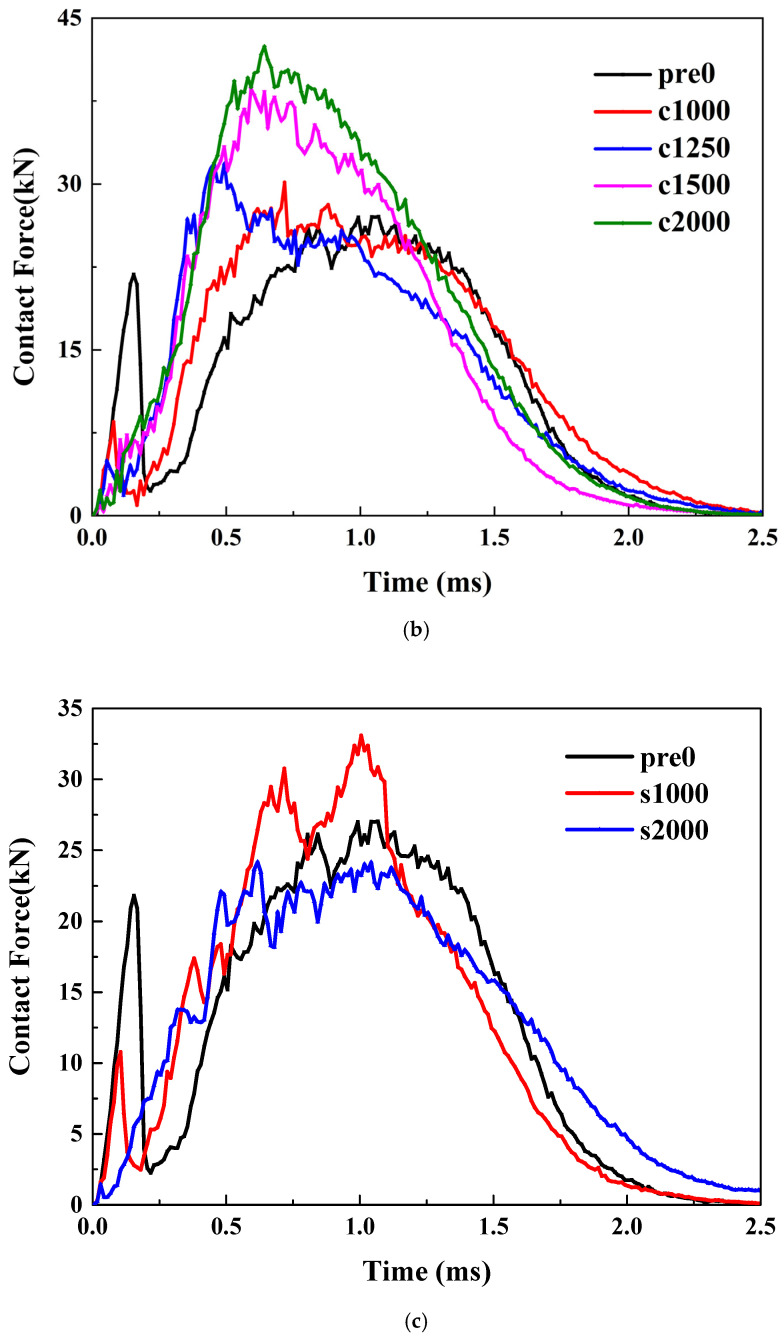
LVI response of thick plate under: (**a**) pretension; (**b**) precompression; (**c**) pre-shear.

Pretension. Unlike thin plates, the peak load remains relatively unchanged when thick plates are subjected to tensile preloads, as can be seen in Figure 5a. However, the trends of the curves differ significantly. A notable feature is that, in the absence of any preload, the load undergoes a process of decreasing and then increasing. As the tensile prestress increases, this decrease almost disappears when the prestress reaches 2000 με.

Precompression. The effect of compressive preload on the impact load of thick plates is highly significant. Firstly, regarding the peak force, compared to the scenario without preload represented by the black curve, when the compressive prestrain reaches 2000 με, the peak load increases by more than 50% (42/27 − 1 ≈ 55%), as demonstrated in Figure 5b. The trend of the curve also shows a notable difference, as the occurrence time of the peak force advances with the increase in compressive preload. Lastly, for the initial rise and subsequent fall observed in the curve’s ascending phase, this phenomenon becomes less pronounced when the compressive prestrain exceeds 1000 με.

Pre-shear. For thick plates, the peak load shown in Figure 5c initially increases and then decreases as the shear prestress increases. This trend is the opposite of what was observed in the case of thin plates mentioned earlier. Regarding the initial decrease followed by an increase in the ascending curve segment, this phenomenon diminishes as shear and load increase. By the time the strain reaches 2000 με, it becomes nearly imperceptible.

The failure in the impact location is dominated by the compressive failure of the matrix, as indicated by Figure 3. Therefore, when in-plane preload is applied, the stress state will change accordingly. For example, compared with the one-directional compression for the no preloading condition, applying a compressive in-plane preloading will result in two-directional compression, which will then reduce the likelihood of crack propagation. Under these conditions, the material demonstrates enhanced resistance to failure. In extreme scenarios, when a material is subjected to hydrostatic pressure—meaning it experiences uniform compressive stress from all directions—it is highly resistant to failure. This is because hydrostatic pressure eliminates tensile stress components, preventing cracks from propagating. For instance, objects in deep-sea environments endure immense hydrostatic pressure but do not easily fracture.

#### 3.2.3. Effects of Impact Energy

**Figure 6:**
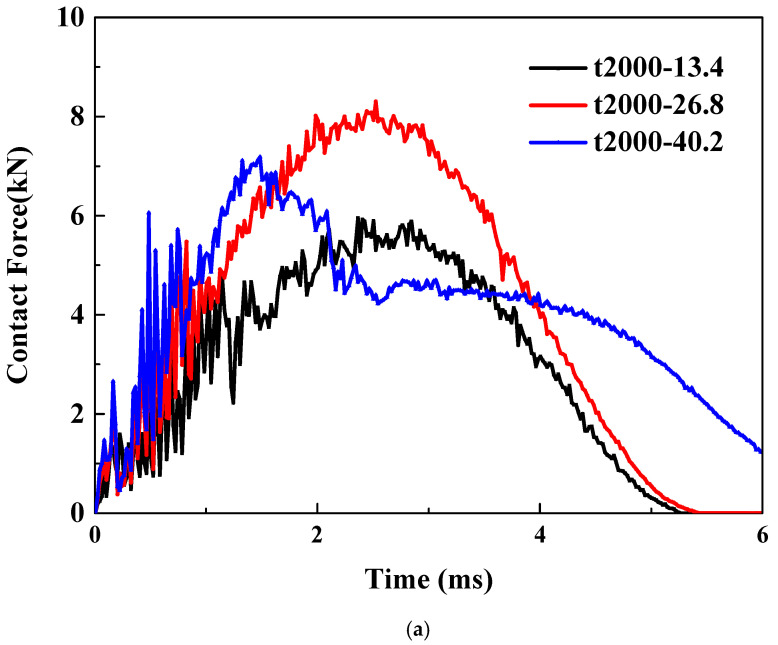

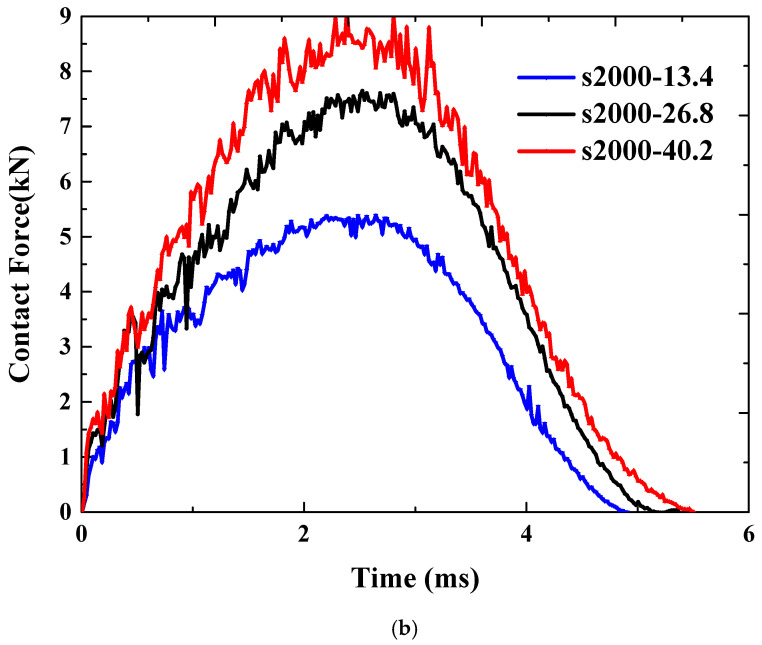
Effects of impact energy on LVI response of thin plate under: (**a**) pretension; (**b**) pre-shear.

The pretension and pre-shear conditions are considered for the thin plate of Type-A. As illustrated in Figure 6a, under a tensile strain of 2000 με, the load–time curves exhibit significant differences when subjected to three distinct impact energies. As the impact energy increases from a relatively low 13.4 J (the case denoted by black curve) to 26.8 J (the case denoted by red curve), the maximum impact energy shows an approximate 50% increase, while the overall trend of the curves remains consistent. However, under a higher impact energy of 40.2 J, the altered trend of the curve suggests a different failure mode. The comparison under a shear strain of 2000 με is shown in Figure 6b. The trend of the curves remains consistent, indicating no change in the failure mode. As the impact energy increases, the peak impact force also rises. Upon closer examination, it can be observed that the rate of increase gradually slows down.

**Figure 7:**
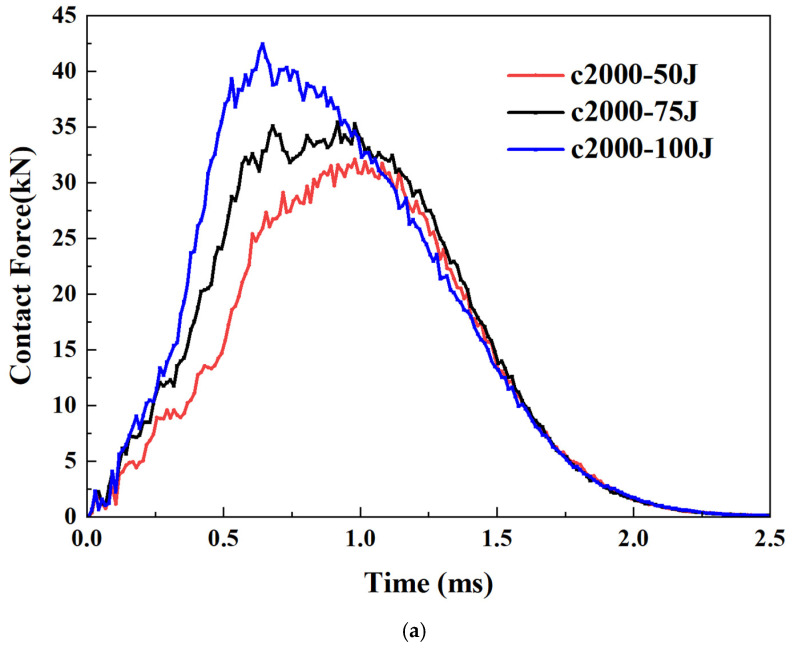

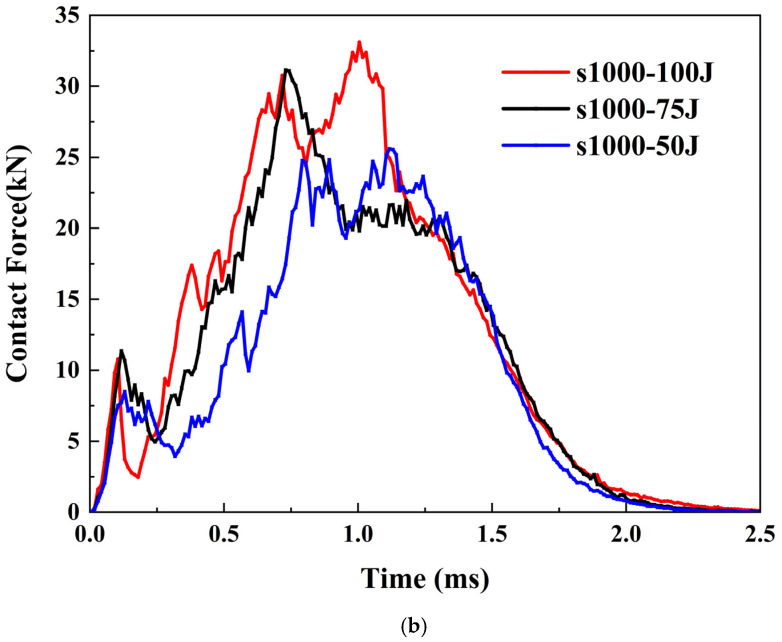
Effects of impact energy on LVI response of thick plate under: (**a**) precompression; (**b**) pre-shear.

Meanwhile, for the thick plate of Type-B, the precompression and pre-shear are considered. Under a compression strain of 2000 με, as presented in Figure 7a, the curves under three different impact energies exhibit consistent trends, indicating the same failure mode. As the energy increases, the curves also rise accordingly. Across the three energy ranges being compared, the magnitude of the peak increase appears to grow progressively. Specifically, an increment of 25 joules results in a more pronounced enhancement when increasing from 70 to 100 joules compared to other ranges. Finally, the impact energy effects were compared under the condition of 1000 με shear strain. As shown in Figure 7b, the descending segments exhibit minimal differences, whereas the ascending segments demonstrate a clear upward trend as the energy increases.

#### 3.2.4. Effects of Impact Angle on the Contact Force

**Figure 8:**
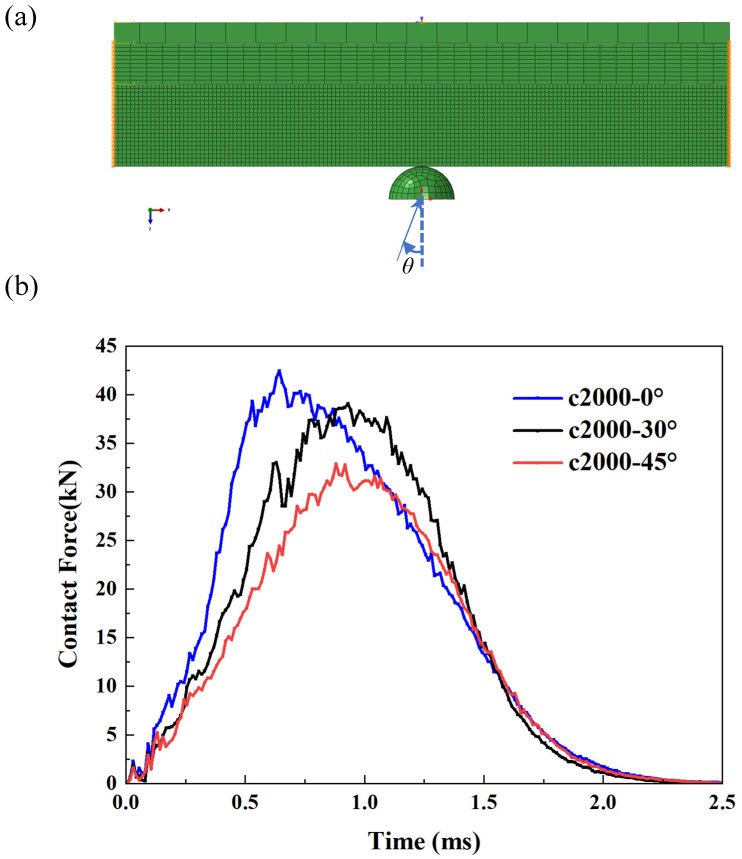
Effects of impact angle on LVI response of thick plate under precompression. (**a**) explanation of impact angle; (**b**) comparison of contact force.

In this final section, we present the results of the thick plate under the influence of compressive prestress (2000 με) when subjected to impacts at various angles. In other words, all the previous results pertain to normal impacts with an impact angle of zero. As illustrated in Figure 8, two cases of oblique impacts are compared with the normal impact scenario. As can be observed in the figure, the load curve under normal impact is the highest, indicating that this represents the most hazardous operating condition.

## 4. Concluding Remarks

This study aims to provide a comprehensive understanding of how preloading and thickness affect the impact resistance of hybrid composite laminates, which is crucial for the design of robust and reliable composite structures in railway vehicles. By integrating experimental validation with finite element analysis, this research contributes to the development of advanced composite materials and their effective application in the railway industry. The simulation results were validated through experimental load–time curves and post-impact CT images. Moreover, key findings are shown in the following Table 4. Effects of preloading on LVI response of thin plates are related to the bending and failure in the back ply. Meanwhile, for thick plates, the existence of preloading will change the stress state in the front ply, which dominates the failure of plate.

## Figures and Tables

**Table 1:** Laminate layups.

(a) Type A: thin plate			
**Layer No.**	**Material**	**Thickness (mm)**	**Orientation (°)**
1	WOVN	1	±45
2	UDLM	0.6	0
3	UDLM	0.4	0
4	UDLM	0.4	0
5	UDLM	0.6	0
6	WOVN	1	±45
(b) Type B: thick plate			
**Group No.**	**Material**	**Thickness (mm)**	
1	WOVN	1	
2	UDLM	3	
3	WOVN	1	
4	UDLM	3	
5	WOVN	1	
6	UDLM	2	
7	WOVN	1	
8	UDLM	3	
9	WOVN	1	
10	UDLM	3	
11	WOVN	1	
12	Core-1	5	
13	Core-2	2	
14	Core-3	3	

**Table 2:** Material properties of composites.

(a) Elastic									
	**Elastic Modulus**			**Poisson’s Ratio**			**Shear Modulus**		
**Material**	**E11** **(GPa)**	**E22** **(GPa)**	**E33** **(GPa)**	**ν12**	**ν13**	**ν23**	**G12** **(GPa)**	**G13** **(GPa)**	**G23** **(GPa)**
UDLM	119.8	10.5	10.5	0.3	0.3	0.48	5.2	5.2	3.7
WOVN	53.7	53.7	11.7	0.033	0.33	0.33	20.7	4.0	4.0
(b) Strength (MPa)									
	**Tensile**			**Compressive**			**Shear**		
**Material**	**F1t**	**F2t**	**F3t**	**F1c**	**F2c**	**F3c**	**τ12**	**τ13**	**τ23**
UDLM	2470	85	85	1062	275	275	89	89	89
WOVN	565	565	49.5	420	420	220	200	200	200

**Table 3:** Elastic properties of core layers.

**Material**	**E11** **(** **MPa)**	**E22** **(** **MPa)**	**E33** **(** **MPa)**	**ν12**	**ν13**	**ν23**	**G12** **(** **MPa)**	**G13** **(** **MPa)**	**G23** **(** **MPa)**
Core-1	85,349.3	25,348.5	7600	0.338	0.338	0.338	10,604.3	3059	3059
Core-2	39,317.6	60,501.3	7600	0.201	0.201	0.201	14,686.9	3059	3059
Core-3	60,035.6	37,932.2	7600	0.242	0.242	0.242	11,701.1	3059	3059

**Table 4:** Summary of effects on the impact response.

Plate	Loading	Peak Load	Peak Timing	Initial Decrease
Thin (4 mm)	Pretensile	Higher	Not significant	N/A
	Precompressive	Not significant	Postponed	N/A
	Preshear	Lower, then higher	Not significant	N/A
	Impact energy-t2000	Higher, then lower	Not significant	N/A
	Impact energy-s1000	Higher	Not significant	N/A
Thick (30 mm)	Pretensile	Not significant	Not significant	Diminishes
	Precompressive	Higher	Advanced	Diminishes
	Preshear	Higher, then lower	Advanced	Diminishes
	Impact energy-c2000	Higher	Advanced	Not significant
	Impact energy-s2000	Higher	Advanced	Enhanced
	Impact angle-c2000	Lower	Postponed	Not significant

## Data Availability

The data that support the findings of this study are not publicly available due to privacy and confidentiality agreements. Data are available from the corresponding author upon reasonable request.
